# RNAi-mediated knock-down of Dab and Numb attenuate Aβ levels via γ-secretase mediated APP processing

**DOI:** 10.1186/2047-9158-1-8

**Published:** 2012-03-22

**Authors:** Zhongcong Xie, Yuanlin Dong, Uta Maeda, Weiming Xia, Rudolph E Tanzi

**Affiliations:** 1 Department of Neurology, Genetics and Aging Research Unit, MassGeneral Institute for Neurodegenerative Disease, Massachusetts General Hospital and Harvard Medical School, Charlestown, MA 02129-2060, USA; 2 Department of Anesthesia and Critical Care, Geriatric Anesthesia Research Unit, Massachusetts General Hospital and Harvard Medical School, Charlestown, MA 02129-2060, USA; 3 Center for Neurological Diseases, Harvard Institute of Medicine and Harvard Medical School, Boston, MA 02115, USA; 4 Graduate student, Department of Psychology, University of Southern California, Los Angeles, CA 90089, USA

## Abstract

Amyloid-β-protein (Aβ), the key component of senile plaques in Alzheimer's disease (AD) brain, is produced from amyloid precursor protein (APP) by cleavage of β-secretase and then γ-secretase. APP adaptor proteins with phosphotyrosine-binding (PTB) domains, including Dab (gene: *DAB*) and Numb (gene: *NUMB*), can bind to and interact with the conserved YENPTY-motif in the APP C-terminus. Here we describe, for the first time, the effects of RNAi knock-down of Dab and Numb expression on APP processing and Aβ production. RNAi knock-down of Dab and Numb in H4 human neuroglioma cells stably transfected to express either FL-APP (H4-FL-APP cells) or APP-C99 (H4-APP-C99 cells) increased levels of APP-C-terminal fragments (APP-CTFs) and lowered Aβ levels in both cell lines by inhibiting γ-secretase cleavage of APP. Finally, RNAi knock-down of APP also reduced levels of Numb in H4-APP cells. These findings suggest that pharmacologically blocking interaction of APP with Dab and Numb may provide novel therapeutic strategies of AD. The notion of attenuating γ-secretase cleavage of APP via the APP adaptor proteins, Dab and Numb, is particularly attractive with regard to therapeutic potential, given that side effects of γ-secretase inhibition owing to impaired proteolysis of other γ-secretase substrates, e.g. Notch, might be avoided.

## Introduction

Amyloid-β-protein (Aβ), the key component of senile plaques in Alzheimer's disease (AD) neuropathology, was first isolated from meningovascular amyloid deposits in AD and Down's syndrome [1,2], and has also been reported to be the subunit of the plaque amyloid [2-4]. The current amyloid hypothesis of AD states that the imbalance between Aβ generation and Aβ clearance is the basis of AD neuropathogenesis. Aβ is generated from amyloid precursor protein (APP). Specifically, APP is first hydrolyzed by β-secretase to generate a 99-residue membrane-associated C-terminus fragment (APP-C99) [5-8]. APP-C99 is further cleaved to release a ~4-kDa peptide, Aβ, and the amyloid precursor protein intracellular domain (AICD). This cleavage is achieved by an unusual form of proteolysis in which the protein is cleaved within the transmembrane domain (at residue +40 or +42) by γ-secretase [9-11]. α-secretase cleaves the majority of APP in the middle of the Aβ region of APP. This cleavage will preclude Aβ generation, lead to the release of a large ectodomain (α-APPs), and leave behind a carboxy-terminus fragment of 83 amino acids (APP-C83) in the membrane. γ-Secretase cleaves APP-C83 to produce p3, an amino-terminally truncated form of Aβ [12,13], [see review in [14]].

The cleavage of the APP cytoplasmic tail by γ-secretase generates AICD, which contains the strongly conserved YENPTY-motif. The YENPTY-sequence is a consensus motif for the binding of adaptor proteins that possess a phosphotyrosine-binding domain (PTB) present in several APP adaptor proteins, such as X11, Fe65, ShcC, Numb, Dab and JIP families [see review in [15]]. We have previously reported that RNAi knock-down of X11α, ShcC and Fe65 in H4 human neuroglioma cells lower Aβ levels [16,17].

Dab (encoded by gene *DAB*), the PTB-containing APP adaptor protein, can bind to and interact with the YENPTY-motif of APP [18,19]. Dab has been reported to function as an adaptor molecule in signal transduction process [20,21]. Numb (encoded by gene *NUMB*) is known to interact via its PTB domain with APP [22,23]. A recent study [24] also suggest that high levels of Notch, another substrate of γ-secretase, can reduce levels of Numb and Numblike.

To date, the effects of reduced expression of Dab and Numb on APP processing and Aβ production, the key components of AD neuropathogenesis, have not been assessed. For this purpose, we established RNAi knock-down of Dab and Numb in H4 human neuroglioma cells overexpressing full-length (FL)-APP (H4-FL-APP cells) and C-99 (H4-APP-C99 cells), and evaluated the effects of RNAi-mediated knock-down of Dab and Numb on APP processing and Aβ levels.

## Experimental procedures

### Cell lines

We employed naïve H4 human neuroglioma (H4) cells and H4 cells stably transfected to express either FL-APP (H4-FL-APP cells) or APP-C99 (H4-APP-C99 cells). Peptide APP-C99 is the product of β-secretase, which therefore contains α- and γ-, but not β-cleavage sites. The H4-APP-C99 cells provide a valid system to assess whether any effects on APP processing are dependent on γ-secretase-mediated APP processing and independent of β-secretase-mediated APP processing. All cell lines were cultured in DMEM (high glucose) containing 9% heat-inactivated fetal calf serum, 100 units/ml penicillin, 100 μg/ml streptomycin, and 2 mM L-glutamine. Stably transfected H4 cells were additionally supplemented with 200 μg/ml G418.

### RNAi treatment

Small interfering RNA (siRNA) duplex was designed and obtained from Qiagen against human *NUMB*, the gene encoding Numb (5'- CAGCCTCTTGACCTCGGATAA-3'). Dab siRNA duplex for *DAB*, the gene encoding Dab, was designed and obtained from Dharmacon research, Inc. (Lafayette, CO 80026) (5'-NNAGGUCAGGAUCGCAGUGAA-3'). Scrambled siRNA (AATTCTCCGAACGTGTCACGT) was obtained from Qiagen and was used as the control siRNA. siRNAs were transfected into cells by using electroporation (AMAXA, Gaithersburg, MD). We mixed 1 million cells, 100 ul AMAXA electroporation transfection solution and 10 ul 20 uM siRNA together, then we employed C-9 program in the AMAXA electroporation device for the cell transfection. We chose 20 uM siRNA of *NUMB *and *DAB *in current studies because our previous studies have shown that 20 uM siRNA of other APP adaptor protein genes, including *APBA1 *[17], *SHC3 *[16] and *APBB1 *[16], can affect APP processing and reduce Aβ levels. The transfected cells then were placed in one well of a six-well plate containing 1.5 ml cell culture media. The cells were harvested 48 hours after siRNA treatments.

### Cell lysis and protein amount quantification

Cell pellets were detergent-extracted on ice using immunoprecipitation buffer (10 mM Tris-HCl, pH 7.4, 150 mM NaCl, 2 mM EDTA, 0.5% Nonidet P-40) plus protease inhibitors (1 μg/ml aprotinin, 1 μg/ml leupeptin, 1 μg/ml pepstatin A). The lysates were collected, centrifuged at 12,000 rpm for 10 min, and quantified for total proteins by the BCA protein assay kit (Pierce, Iselin, NJ).

### Western blot analysis of APP processing

Western blot analysis was performed as described by Xie et al. [25]. Briefly, 40 μg of total protein of each sample was subjected to SDS-polyacrylamide gel electrophoresis using 4-20% gradient Tris/glycine gels under reducing conditions (Invitrogen, Carlsbad, CA). Next, proteins were transferred to a polyvinylidene difluoride membrane (Bio-Rad, Hercules, CA) using a semi-dry electrotransfer system (Amersham Biosciences, San Francisco, CA). Nonspecific proteins were blocked using 5% non-fat dry milk in TBST for 1.5 h. Blots were then incubated with a primary antibody, followed by a secondary antibody (horseradish peroxidase-conjugated anti-rabbit antibody 1:10,000; Pierce, New York, NY). Blots were washed with TBST for 30 min between steps. Antibody Dab (1:1,000, Novus Biologicals, Littleton, CO) was used to recognize Dab (35 kDa), antibody Numb (1:1,000, Abcam, Cambridge, MA) was used to detect Numb (75 kDa). Antibody A8717 (1:1,000, Sigma, St. Louis, MO) was used to visualize FL-APP (110 kDa), APP-C83 (12 kDa) and APP-C99 (10 kDa) in the Western blot analysis. The intensity of signals was analyzed using an image program (NIH Image 1.62). We first used the levels of β-actin to normalize the levels of Numb, Dab, FL-APP and APP-CTFs (e.g., determining the ratio of Numb amount to β-actin amount) to control for loading differences in total protein amounts. We then presented the changes in the protein levels of Numb, Dab, FL-APP, APP-C99 and APP-C83 in the cells treated with Numb or Dab siRNA as the percentage of those in the cells treated with control siRNA.

### Quantitation of Aβ using Sandwich ELISA assay

Following the treatment with control siRNA, Numb siRNA or Dab siRNA, conditioned media was collected, and secreted Aβ was measured with a Sandwich ELISA assay by the Aβ ELISA Core Facility at the Center for Neurological Diseases, Harvard Institute of Medicine, Harvard Medical School, Boston, Massachusetts, as described by Xie et al. [16]. Specifically, 96-well plates were coated with mouse monoclonal antibodies (mAb) specific to Aβ40 (2 G3). Following blocking with Block Ace, wells were incubated overnight at 4°C with test samples of conditioned cell culture media, and then an anti-Aβ (α-Aβ-HR1) conjugated to horseradish peroxidase was added. Plates were then developed with TMB reagent and well absorbance was measured at 450 nm. Aβ levels in test samples were determined by comparison with the signal from unconditioned media spiked with known quantities of Aβ40.

### Statistics

ANOVA with repeated measurements was employed to compare the difference from the control group. P-values less than 0.05 were considered statistically significant.

## Results

### RNAi knock-down of Dab increased APP-CTFs levels and decreased Aβ levels in H4-FL-APP cells

**Figure 1:**
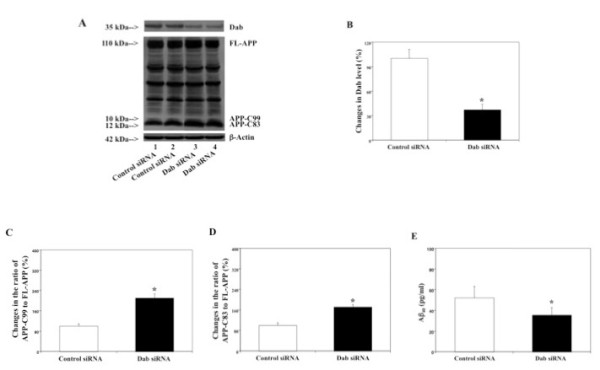
**Effects of RNAi knock-down of Dab on APP processing and Aβ levels in H4-FL-APP cells**. In H4-FL-APP cells, Dab siRNA treatment decreases protein levels of Dab, increases protein levels of APP-C83 and APP-C99, and decreases Aβ levels. A. APP processing in Western blot analyses. Dab immunoblotting shows reductions in protein levels of Dab in the cells treated with Dab siRNA (columns 3 and 4) as compared to control siRNA (columns 1 and 2). FL-APP immunoblotting reveals that there is no significant difference in the protein levels of FL-APP in the cells treated with control siRNA or Dab siRNA. APP-CTFs immunoblotting shows increases in protein levels of APP-C99 and APP-C83 in the cells treated with Dab siRNA (columns 3 and 4) as compared to control siRNA (columns 1 and 2). B. Protein levels of Dab assessed by quantifying Dab in the Western blot. Dab siRNA treatment (black bar) significantly decreases protein levels of Dab as compared to control siRNA treatment (white bar) (*p < 0.05), normalized to β-actin. C. APP processing assessed by quantifying ratio of APP-C99 to FL-APP in the Western blot. Dab siRNA treatment (black bar) significantly increases ratio of APP-C99 to FL-APP as compared to control siRNA treatment (white bar) (*p < 0.05), normalized to β-actin. D. APP processing assessed by quantifying ratio of APP-C83 to FL-APP in the Western blot. Dab siRNA treatment (black bar) significantly increases ratio of APP-C83 to FL-APP as compared to control siRNA treatment (white bar) (*p < 0.05), normalized to β-actin. E. Effects of RNAi knock-down of Dab on Aβ levels in H4-FL-APP cells. Dab siRNA treatment (black bar) decreases Aβ40 levels as compared to control siRNA treatment (white bar) (*p < 0.05).

We first established conditions under which RNAi knock-down of Dab significantly reduced protein levels of Dab in H4-FL-APP cells. The cells were harvested 48 hours after being transfected with either control siRNA or Dab siRNA, and were subjected to Western blot analyses in which antibody Dab was used to visualize Dab levels. Immunoblotting for Dab revealed a visible reduction in Dab levels following Dab siRNA treatment as compared to control siRNA treatment (Figure 1A). Dab siRNA treatment significantly reduced Dab levels by 44% (normalized to β-actin) as compared to control siRNA treatment (Figure 1B, *p < 0.05).

Next, we assessed effects of RNAi-mediated knock-down of Dab on APP processing in H4-FL-APP cells by measuring protein levels of FL-APP, APP-C99 and APP-C83 following Dab or control siRNA treatments. 48 hours after transfection of Dab siRNA or control siRNA, the cells were harvested and subjected to Western blot analyses in which antibody A8717 was used to detect FL-APP, APP-C99 and APP-C83. Immunoblot analysis of APP-CTFs revealed increases in levels of APP-C99 and APP-C83 in the cells treated with Dab siRNA as compared to the cells treated with control siRNA (Figure 1A). Meanwhile, no significant differences in FL-APP levels were observed between Dab siRNA- and control siRNA-treated cells. Quantification of FL-APP, APP-C99 and APP-C83 (normalized to β-actin) revealed that Dab siRNA treatment led to a 207% increase in ratio of APP-C99 to FL-APP (Figure 1C, *p < 0.05), and a similar (176%) increase in ratio of APP-C83 to FL-APP (Figure 1D, *p < 0.05), as compared to control siRNA treatment. Note the different parts of Western blot representing Dab were obtained from same samples of the experiments.

Next, we assessed effects of Dab siRNA on Aβ levels in conditioned media. 48 hours after treatment with control siRNA or Dab siRNA, we measured secreted Aβ40 levels in conditioned cell culture media. Dab siRNA treatment significantly decreased Aβ40 levels as compared to control siRNA treatment (Figure 1E, *p < 0.05): 34 pg/ml (Dab siRNA) versus 57 pg/ml (control siRNA). Collectively, these data indicate that RNAi knock-down of Dab increases levels of APP-C99, APP-C83, and decreases secreted Aβ in H4-FL-APP cells, in a manner similar to that of γ-secretase inhibitor treatment.

### RNAi knock-down of Numb increased APP-CTFs levels and decreased Aβ levels in H4-FL-APP cells

**Figure 2:**
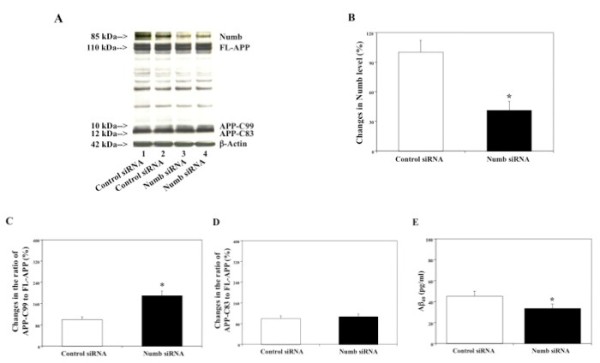
**Effects of RNAi knock-down of Numb on APP processing and Aβ levels in H4-FL-APP cells**. In H4-FL-APP cells, Numb siRNA treatment decreases protein levels of Numb, increases protein levels of APP-C99, and decreases Aβ levels. A. APP processing in Western blot analyses. Numb immunoblotting shows reductions in protein levels of Numb in the cells treated with Numb siRNA (columns 3 and 4) as compared to control siRNA (columns 1 and 2). FL-APP immunoblotting reveals that there is no significant difference in protein levels of FL-APP in the cells treated with control siRNA or Numb siRNA. APP-CTFs immunoblotting shows increases in protein levels of APP-C99 in the cells treated with Numb siRNA (columns 3 and 4) as compared to control siRNA (columns 1 and 2). B. Protein levels of Numb assessed by quantifying Numb in the Western blot. Numb siRNA treatment (black bar) significantly decreases protein levels of Numb as compared to control siRNA treatment (white bar) (*p < 0.05), normalized to β-actin. C. APP processing assessed by quantifying ratio of APP-C99 to FL-APP in the Western blot. Numb siRNA treatment (black bar) significantly increases ratio of APP-C99 to FL-APP as compared to control siRNA treatment (white bar) (*p < 0.05), normalized to β-actin. D. APP processing assessed by quantifying ratio of APP-C83 to FL-APP in the Western blot. Numb siRNA treatment (black bar) does not significantly increase ratio of APP-C83 to FL-APP as compared to control siRNA treatment (white bar), normalized to β-actin. E. Effects of RNAi knock-down of Numb on Aβ levels in H4-FL-APP cells. Numb siRNA treatment (black bar) decreases Aβ40 levels as compared to control siRNA treatment (white bar) (*p < 0.05).

We next assessed effects of RNAi knock-down of Numb, another APP adapter molecule, on APP processing and Aβ levels in H4-FL-APP cells. We first established conditions under which Numb siRNA treatment reduced protein levels of Numb in H4-FL-APP cells. The cells were harvested 48 hours after being transfected with either control siRNA or Numb siRNA, and were subjected to Western blot analyses with Numb antibody to measure protein levels of Numb. Numb immunoblotting revealed a 54% reduction in protein levels of Numb following Numb siRNA treatment as compared to control siRNA treatment (Figure 2A, B, *p < 0.05).

We then assessed effects of RNAi-mediated knock-down of Numb on APP processing in H4-FL-APP cells by measuring levels of FL-APP, APP-C99 and APP-C83 following Numb siRNA or control siRNA treatments. 48 hours after transfection of Numb siRNA or control siRNA, the cells were harvested and subjected to Western blot analyses with antibody A8717 to detect FL-APP, APP-C99, and APP-C83. Levels of APP-C99 and APP-C83 were increased in the cells treated with Numb siRNA versus control siRNA (Figure 2A). Meanwhile, no significant differences in FL-APP levels were observed for Numb siRNA versus control siRNA-treated cells (Figure 2A). Numb siRNA treatment led to a 202% increase in ratio of APP-C99 to FL-APP (Figure 2C, *p < 0.05), but only a mild (122%) increase in ratio of APP-C83 to FL-APP (Figure 2D, N.S.), normalized to β-actin, as compared to control siRNA treatment.

Next, we measured secreted Aβ levels in conditioned cell culture media. 48 hours after treatment with control siRNA or Numb siRNA in H4-FL-APP cells, Numb siRNA decreased Aβ40 levels: 34 pg/ml for Numb siRNA versus 48 pg/ml for control siRNA (Figure 2E, *p < 0.05). Collectively, these data indicate that RNAi knock-down of Numb also affects APP processing and Aβ production in a manner similar to that of γ-secretase inhibitor treatment in H4-FL-APP cells.

As discussed in prior section, β-secretase cleaves FL-APP to produce APP-C99, which can be cleaved by γ-secretase to produce Aβ. Therefore, changes in APP processing and Aβ production following treatments of Dab siRNA and Numb siRNA could be due to alterations in either β-secretase or γ-secretase activities. In the following experiments, we set out to determine the extent to which the alterations in APP processing and Aβ production following RNAi knock-down of Dab or Numb were independent of β-secretase-mediated APP processing and dependent on γ-secretase-mediated APP processing, employing H4 cells over-expressing APP-C99 (H4-APP-C99 cells).

### RNAi knock-down of Dab increased APP-CTFs levels and decreased Aβ levels in H4-APP-C99 cells

We employed H4-APP-C99 cells in order to determine whether RNAi knock-down of Dab-induced alterations in APP processing and Aβ levels were independent of β-secretase-mediated APP processing. As described in the prior section, APP-C99 is the product of β-secretase and harbors α- and γ-cleavage, but not β-cleavage sites, therefore H4-APP-C99 cells provide a valid system to assess whether any effects on APP processing are dependent on γ-secretase-mediated APP processing and independent of β-secretase-mediated APP processing.

**Figure 3:**
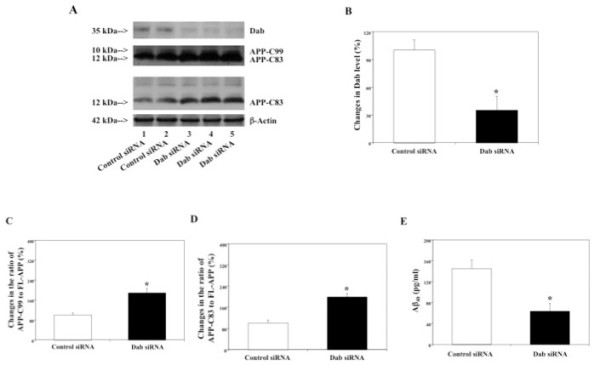
**Effects of RNAi knock-down of Dab on APP processing and Aβ levels in H4-APP-C99 cells**. In H4-APP-C99 cells, Dab siRNA treatment decreases protein levels of Dab, increases protein levels of APP-C83 and APP-C99, and decreases Aβ levels. A. APP processing in Western blot analyses. Dab immunoblotting shows reductions in protein levels of Dab in the cells treated with Dab siRNA (columns 3 to 5) as compared to the cells treated with control siRNA (columns 1 and 2). APP-CTFs immunoblotting shows increases in protein levels of APP-C99 and APP-C83 in the cells treated with Dab siRNA (columns 3 to 5) as compared to control siRNA (columns 1 and 2). The blot showing the band of APP-C83 only is the same blot with less exposure time in developing the film. B. Dab protein levels assessed by quantifying Dab in the Western blot. Dab siRNA treatment significantly decreases protein levels of Dab as compared to control siRNA treatment (*p < 0.05), normalized to β-actin. C. APP processing assessed by quantifying ratio of APP-C99 to endogenous FL-APP in the Western blot. Dab siRNA treatment significantly increases ratio of APP-C99 to FL-APP as compared to control siRNA treatment (*p < 0.05), normalized to β-actin. D. APP processing assessed by quantifying ratio of APP-C83 to endogenous FL-APP in the Western blot. Dab siRNA treatment significantly increases ratio of APP-C83 to FL-APP as compared to control siRNA treatment (*p < 0.05), normalized to β-actin. E. Effects of RNAi knock-down of Dab on Aβ levels in H4-APP-C99 cells. Dab siRNA treatment decreases Aβ40 levels as compared to control siRNA treatment (*p < 0.05).

48 hours after transfection of H4-APP-C99 cells with Dab siRNA or control siRNA, the cells were harvested and subjected to Western blot analyses in which Dab antibody was used to detect Dab. Dab immunoblotting revealed a significant reduction in protein levels of Dab in the cells treated with Dab siRNA as compared to the cells treated with control siRNA (Figure 3A). There was no significant difference in amount of β-actin in control siRNA- and Dab siRNA-treated cells. Quantification of Dab in the Western blot, normalized to β-actin, showed that Dab siRNA treatment caused a 65% reduction in protein levels of Dab (Figure 3B, *p < 0.05).

We next assessed effects of RNAi-mediated knock-down of Dab on APP processing. 48 hours after transfection with Dab siRNA or control siRNA, the cells were harvested and subjected to Western blot analyses with antibody A8717. Dab siRNA treatment did not alter endogenous levels of FL-APP as compared to control siRNA treatment (data not shown). APP-CTFs immunoblotting revealed visible increases in protein levels of both APP-C99 and APP-C83 in the H4-APP-C99 cells treated with Dab siRNA, compared to control siRNA (Figure 3A). There was no significant difference in amount of β-actin in the control siRNA- or Dab siRNA-treated H4-APP-C99 cells. Quantification of FL-APP, APP-C99 and APP-C83 revealed that Dab siRNA treatment led to a 188% increase in ratio of APP-C99 to FL-APP (Figure 3C, *p < 0.05) and a 199% increase in ratio of APP-C83 to FL-APP (Figure 3D, *p < 0.05), as compared to control siRNA treatment.

We then measured secreted Aβ levels in conditioned cell culture media 48 hours after treatment with either control siRNA or Dab siRNA in H4-APP-C99 cells. Dab siRNA decreased Aβ levels as compared to control siRNA treatment: 63.5 pg/ml (Dab siRNA treatment), 144.5 pg/ml (control siRNA treatment) (Figure 3E, *p < 0.05). These findings suggest that effects of RNAi knock-down of Dab on APP processing and Aβ generation are dependent on γ-secretase- and independent of β-secretase-mediated cleavage of APP.

### RNAi knock-down of Numb increased APP-CTFs levels and decreased Aβ levels in H4-APP-C99 cells

**Figure 4:**
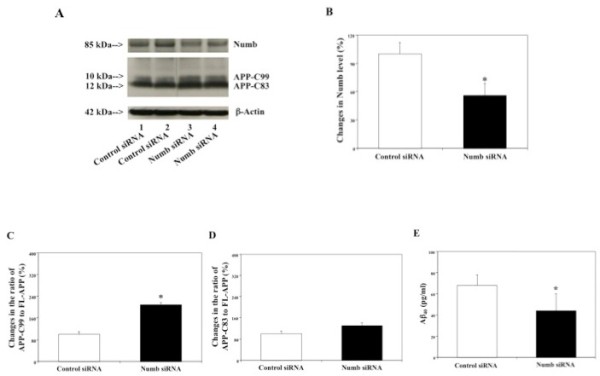
**Effects of RNAi knock-down of Numb on APP processing and Aβ levels in H4-APP-C99 cells**. In H4-APP-C99 cells, Numb siRNA treatment decreases protein levels of Numb, increases protein levels of APP-C99 and decreases Aβ levels. A. APP processing in Western blot analyses. Numb immunoblotting shows reductions in protein levels of Numb in the cells treated with Numb siRNA (columns 3 and 4) as compared to control siRNA (columns 1 and 2). APP-CTFs immunoblotting shows increases in protein levels of APP-C99 in the cells treated with Numb siRNA (columns 3 and 4) as compared to control siRNA (columns 1 and 2). B. Numb protein levels assessed by quantifying Numb in the Western blot. Numb siRNA treatment significantly decreases protein levels of Numb as compared to control siRNA treatment (*p < 0.05), normalized to β-actin. C. APP processing assessed by quantifying ratio of APP-C99 to endogenous FL-APP in the Western blot. Numb siRNA treatment significantly increases ratio of APP-C99 to FL-APP as compared to control siRNA treatment (*p < 0.05), normalized to β-actin. D. APP processing assessed by quantifying ratio of APP-C83 to endogenous FL-APP in the Western blot. Numb siRNA treatment does not significantly increase ratio of APP-C83 to FL-APP as compared to control siRNA treatment (*p < 0.05), normalized to β-actin. E. Effects of RNAi knock-down of Numb on Aβ levels in H4-APP-C99 cells. Numb siRNA treatment decreases Aβ40 levels as compared to control siRNA treatment (*p < 0.05).

Next, we asked whether alterations in APP processing and Aβ levels induced by RNAi knock-down of Numb were also independent of β-secretase-mediated APP processing. For this purpose, we set out to determine effects of RNAi knock-down of Numb on APP processing and Aβ levels in H4-APP-C99 cells. We first established RNAi knock-down of Numb in H4-APP-C99 cells by showing that RNAi knock-down of Numb reduced protein levels of Numb in H4-APP-C99 cells (Figure 4A, B, a 46% reduction, *p < 0.05).

We then assessed effects of RNAi knock-down of Numb on levels of APP-C83, APP-C99 and Aβ in H4-APP-C99 cells. 48 hours after transfection with Numb siRNA or control siRNA, the cells were harvested and subjected to Western blot analyses with antibody A8717. Numb siRNA treatment increased protein levels of both APP-C99 and APP-C83 in H4-APP-C99 cells, without altering endogenous FL-APP levels (data not shown), as compared to control siRNA treatment (Figure 4A). Quantification of the Western blot revealed that Numb siRNA treatment led to a 209% increase in the ratio of APP-C99 to FL-APP (Figure 4C, *p < 0.05) and only a 130% increase in ratio of APP-C83 to FL-APP (Figure 3D, N.S.), as compared to control siRNA treatment. Numb siRNA treatment decreased secreted Aβ levels as compared to control siRNA treatment in H4-APP-C99 cells: 44 pg/ml (Numb siRNA treatment) versus 68 pg/ml (control siRNA treatment) (Figure 4E, *p < 0.05). These findings suggest that RNAi knock-down of Numb may affect APP processing and Aβ production at least partially by inhibiting γ-secretase-, but not β-secretase-, mediated cleavage of APP, a manner similar to that of γ-secretase inhibitors.

### RNAi knock-down of APP decreased Numb levels in H4-APP cells

**Figure 5:**
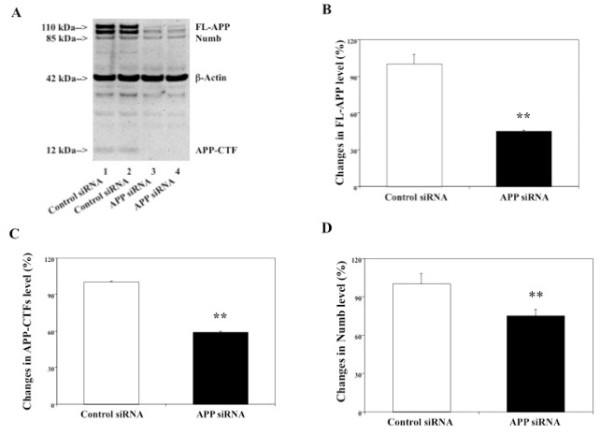
**Effects of RNAi knock-down of APP on levels of Numb in H4-APP cells**. In H4-APP cells, APP siRNA treatment decreases protein levels of Numb. A. Levels of FL-APP, APP-CTFs and Numb in Western blot analyses. APP immunoblotting shows reductions in protein levels of FL-APP, APP-CTFs in the cells treated with APP siRNA (columns 3 and 4) as compared to that treated with control siRNA (columns 1 and 2). Numb immunoblotting shows reductions in protein levels of Numb in the cells treated with Numb siRNA (columns 3 and 4) as compared to control siRNA (columns 1 and 2). B. FL-APP levels assessed by quantifying FL-APP in the Western blot. APP siRNA treatment significantly decreases protein levels of FL-APP as compared to control siRNA (**p < 0.01), normalized to β-actin. C. APP-CTFs levels assessed by quantifying APP-CTFs in the Western blot. APP siRNA treatment significantly decreases protein levels of APP-CTFs as compared to control siRNA (**p < 0.01), normalized to β-actin. D. Numb protein levels assessed by quantifying Numb in the Western blot. APP siRNA treatment significantly decreases protein levels of Numb as compared to control siRNA treatment (*p < 0.05), normalized to β-actin.

A recent study by Chapman et al. [24] showed that high levels of Notch can decrease Numb and Numblike. Both APP and Notch are substrates of γ-secretase, we therefore assessed effects of APP on Numb levels in H4-APP cells. 48 hours after transfection with APP siRNA or control siRNA, the cells were harvested and subjected to Western blot analyses with antibodies anti-Numb and A8717. We first showed that APP siRNA treatment decreased protein levels of both FL-APP and APP-CTFs (Figure 5A), suggesting that the APP siRNA treatment can reduce levels of APP (both FL-APP and APP-CTFs) in H4-APP cells. Then we were able to show that the same APP siRNA treatment also reduced levels of Numb in H4-APP cells (Figure 5A). Quantification of the Western blot revealed that APP siRNA treatment led to a 55% (Figure 5B, **p < 0.01), 41% (Figure 5C, **p < 0.01) and 25% (Figure 5D, **p < 0.01) reduction in levels of FL-APP, APP-CTFs and Numb, respectively, as compared to control siRNA treatment. These findings suggest that RNAi knock-down of APP may affect Numb metabolism as well.

### Control siRNA affected neither APP processing nor Aβ levels in H4-FL-APP or H4-APP-C99 cells

Finally, We found that control siRNA did not affect APP processing or alter Aβ levels as compared to saline treatment in H4-FL-APP cells or H4-APP-C99 cells (data not shown). These results confirmed that effects of RNAi knock-down of Dab or Numb on APP processing and Aβ levels in our experiments were not due to control siRNA (scrambled siRNA) effects, but owing to reductions in protein levels of Dab and Numb.

## Discussion

Aβ accumulation resulting from imbalance between Aβ generation and clearance in brain is a foundation of AD neuropathogenesis [[1], see review in [26]]. Aβ is produced via serial proteolysis of APP by two proteases, β-secretase and γ-secretase [5-8]. Several APP adaptor proteins [see review in [15]] have previously been shown to affect APP processing and Aβ production following overexpression [27-35]. Recent studies showed that RNAi-mediated knock-down of X11α, ShcC, Fe65 and ARH can also affect APP processing and Aβ levels [16,17,36]. However, effects of RNAi knock-down of other APP adaptor proteins, including Dab and Numb, on APP processing and Aβ production have not been previously reported. Here, we show for the first time that RNAi knock-down of Dab and Numb significantly affects APP processing and Aβ levels.

RNAi knock-down of Dab and Numb caused increases in levels of APP-C99 and APP-C83 in absence of alterations in FL-APP levels, and RNAi for Dab and Numb decreased secreted Aβ levels in H4-FL-APP cells. As described in prior section, β-secretase and α-secretase cleaves FL-APP to produce APP-C99 and APP-C83 respectively, then γ-secretase cleaves APP-C99 and APP-C83 to generate Aβ and p3 respectively, and AICD. Therefore, the observed increases in APP-C99 and APP-C83 levels following Dab or Numb siRNA treatment could be due either to increases in the activities of β-secretase and/or α-secretase, or to decreases in γ-secretase cleavage of APP-C99 and C83. To distinguish these two possibilities, we repeated RNAi knock-down of Dab and Numb experiment in H4 cells over-expressing APP-C99 (H4-APP-C99 cells), the β-secretase cleavage product of APP, and found that Dab or Numb siRNA treatment still increased protein levels of APP-C99 and APP-C83 and decreased levels of secreted Aβ. These data indicate that the observed changes in APP processing and Aβ levels following RNAi knock-down of Dab and Numb are independent of β-secretase-, and most likely are due to inhibition of γ-secretase-mediated cleavage of APP.

One possible explanation for our current results is that Dab and Numb, APP adaptor proteins, are essential for γ-secretase cleavage of APP-C99 to generate Aβ, perhaps via facilitating APP trafficking to the sites where γ-secretase can cleave it. Reductions in levels of Dab and Numb by RNAi knock-down for their encoded genes, *DAB *and *NUMB*, will cause a "break" in APP trafficking to the sites where γ-secretase is located, thereby leading to the accumulation of APP-CTFs (APP-C99 and APP-C83), the substrates of γ-secretase, and reduction in levels of Aβ, the products of γ-secretase. In the future studies, we will assess effects of over-expression and reduction of Dab and Numb on APP trafficking to further test this hypothesis.

To date, many other substrates have been reported to undergo γ-secretase cleavage in addition to APP: Notch, ErbB-4, E-cadherin, the LDL receptor-related protein (LRP), CD44 and nectin-1-α [37-43]. All of these substrates are type I membrane proteins residing at or near cell surface, which undergo ectodomain shedding prior to γ-secretase-like cleavage and release ICDs (intracellular domains) following proteolysis. In our future research, we will determine whether or not RNAi knock-down of Dab and Numb can affect processing of these other γ-secretase substrates. It is also important to determine the potential interactions between Notch signaling with APP adaptor proteins including Numb, Dab, X11α, ShcC and Fe65 in our established cellular model.

Taking together, our findings demonstrate, for the first time, that RNAi-mediated knock-down of Dab and Numb decrease secretion of Aβ, conceivably via inhibiting γ-secretase cleavage of APP. These data, together with those of previous studies, imply that blocking interaction of APP with either Dab or Numb, and perhaps other APP adaptor proteins (e.g., X11α, ShcC and Fe65), could be a novel therapeutical strategy for treating and/or preventing AD by lowering Aβ accumulation.

Increasing evidence suggest a role of Notch signaling pathway in neurodegeneration of adult vertebrate nervous system and in AD neuropathogenesis [44,45]. A recent study showed that overexpressions of APP, APLP1 and APLP2 induce Notch gain-of-function phenotypes in *Drosophila*, suggesting a cross-talk between APP family and Notch [46]. Numb and Dab have been suggested to be mediators of such APP and Notch interactions [46]. Moreover, it has been suggested that pharmacological processing of APP in AD treatment may cause alterations in Notch phenotype through Dab and Numb, thereby contributing to the side effects of the treatment or even to increasing risk of AD [46,47]. Therefore, Dab and Numb could be important targets in the development of therapeutical strategies for AD. Studies to further identify the roles of Dab and Numb in AD neuropathogenesis, including assessment of effects of Dab and Numb on APP processing, Aβ accumulation, synaptic function and apoptosis, are warranted in the future.

A recent study [24] showed that at low levels of Notch signaling, Numb and Numblike can negatively regulate Notch, however, high levels Notch can reduce protein levels of Numb and Numblike. These findings suggest that a reciprocal negative regulation between Notch and Numb/Numblike. In the present study, we have illustrated for the first time that reductions in APP levels can lead to reductions in Numb levels, and reductions in Numb levels can lead to reductions in Aβ levels. Collectively, these findings suggest that APP (and Aβ) and Numb can also have a reciprocal negative regulation, leading to reductions in Aβ levels.

In conclusion, RNAi-mediated knock-down of APP adaptor proteins, Dab and Numb, attenuate γ-secretase-mediated cleavage of APP, leading to decreased Aβ levels. Our findings, together with those of previous studies, suggest that pharmaceutical modulation of APP adaptor proteins, might potentially serve as a novel therapeutic approach to treating and preventing AD. The notion of attenuating γ-secretase cleavage of APP via APP adaptor proteins, such as X11α, ShcC, Fe65, Dab and Numb, is particularly attractive, because interactions with these APP adaptor proteins may bypass unwanted effects of γ-secretase inhibition owing to impaired proteolysis of other γ-secretase substrates. More studies are needed to further investigate these findings and to assess feasibility of such a therapeutic strategy aimed at lowering Aβ levels.

## Abbreviations

AD: Alzheimer's disease; siRNA: Small interfering RNA; RNAi: RNA interference; ANOVA: Analysis of variance; NTF: Amino-terminal fragment; CTF: Carboxyl-terminal fragment; FL: Full-length; APP: Amyloid precursor protein; Aβ: Amyloid-β protein; PTB: Phosphotyrosine binding.

## Competing interests

The authors declare that they have no competing interests.

## Authors' contributions

ZX carried out RNAi interference (RNAi) studies, Western blot analysis, experimental design, data analysis and wrote the manuscript. YD carried out RNAi studies and Western blot analysis. UM carried out cell culture and Western blot studies. WX carried out ELISA measurement of Aβ levels. RT carried out the data analysis and drafted the manuscript. All authors read and approved the final manuscript.
